# Nanocrystalline and Amorphous Calcium Carbonate from Waste Seashells by Ball Milling Mechanochemistry Processes

**DOI:** 10.1021/acs.cgd.3c01007

**Published:** 2023-12-22

**Authors:** Chiara Marchini, Carla Triunfo, Nicolas Greggio, Simona Fermani, Devis Montroni, Andrea Migliori, Alessandro Gradone, Stefano Goffredo, Gabriele Maoloni, Jaime Gómez Morales, Helmut Cölfen, Giuseppe Falini

**Affiliations:** † Department of Chemistry “Giacomo Ciamician”, University of Bologna, via F. Selmi 2, 40126 Bologna, Italy; ‡ Fano Marine Center, viale Adriatico 1/N 61032 Fano, Italy; § Department of Biological, Geological and Environmental Sciences, University of Bologna, via F. Selmi 3, 40126 Bologna, Italy; ∥ Institute for Microelectronics and Microsystems (IMM) − CNR section of Bologna, via P. Gobetti 101, 40129 Bologna, Italy; ⊥ Finproject S.p.A., Plant Ascoli Piceno, Via Enrico Mattei, 1-Zona Ind.le Campolungo, 3100 Ascoli Piceno, Italy; # Laboratorio de Estudios Cristalográficos, Instituto Andaluz de Ciencias de la Tierra (CSIC-UGR), Avda Las Palmeras 4, 18100 Armilla, Granada, Spain; ¶ Department of Chemistry, Physical Chemistry, University of Konstanz, Universitätsstrasse 10, Box 714, D-78457 Konstanz, Germany

## Abstract

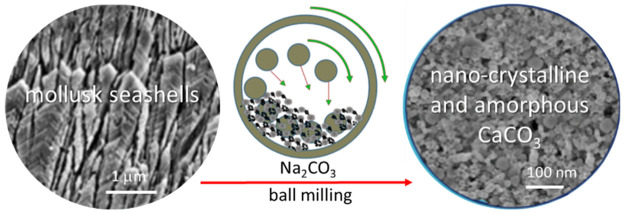

Nanocrystalline calcium carbonate (CaCO_3_) and amorphous CaCO_3_ (ACC) are materials of increasing technological interest. Nowadays, they are mainly synthetically produced by wet reactions using CaCO_3_ reagents in the presence of stabilizers. However, it has recently been discovered that ACC can be produced by ball milling calcite. Calcite and/or aragonite are the mineral phases of mollusk shells, which are formed from ACC precursors. Here, we investigated the possibility to convert, on a potentially industrial scale, the biogenic CaCO_3_ (bCC) from waste mollusk seashells into nanocrystalline CaCO_3_ and ACC. Waste seashells from the aquaculture species, namely oysters (*Crassostrea gigas*, low-Mg calcite), scallops (*Pecten jacobaeus*, medium-Mg calcite), and clams (*Chamelea gallina*, aragonite) were used. The ball milling process was carried out by using different dispersing solvents and potential ACC stabilizers. Structural, morphological, and spectroscopic characterization techniques were used. The results showed that the mechanochemical process produced a reduction of the crystalline domain sizes and formation of ACC domains, which coexisted in microsized aggregates. Interestingly, bCC behaved differently from the geogenic CaCO_3_ (gCC), and upon long milling times (24 h), the ACC reconverted into crystalline phases. The aging in diverse environments of mechanochemically treated bCC produced a mixture of calcite and aragonite in a species-specific mass ratio, while the ACC from gCC converted only into calcite. In conclusion, this research showed that bCC can produce nanocrystalline CaCO_3_ and ACC composites or mixtures having species-specific features. These materials can enlarge the already wide fields of applications of CaCO_3_, which span from medical to material science.

## Abstract 2

Biogenic calcium carbonate from waste mollusc shells can be amorphized in a species-specific mode by ball milling mechanochemistry.

## Introduction

The polymorphs of calcium carbonate (CaCO_3_) include amorphous calcium carbonate (ACC), three anhydrous crystalline phases (calcite, aragonite, and vaterite), and two hydrated phases (monohydrate and hexahydrate). Calcite and aragonite are by far the most common and stable forms, whereas ACC is the least stable polymorph from the viewpoint of thermodynamics.^1^

ACC has been widely found in organisms, where it plays an important role in the biomineralization of crystalline CaCO_3._^2^ It is generally accepted that biogenic ACC can broadly occur in one of two forms: stable or transient.^3^ While the stable form remains noncrystalline, the transient phase can act as a precursor to either calcite or aragonite.

Stable ACC was found in various organisms like mollusk shells,^4^ American lobsters,^5^ coral skeletons,^6^ and so forth. In addition, evidence suggests that calcific biominerals, such as the mollusk nacre^7^ and the urchin’s spine,^8^ are formed from an amorphous precursor.

The popular involvement of ACC in biological processing organisms,^9,10^ usually via complex nonclassical crystallization pathways,^11^ has aroused interest in the scientific community. In recent years, a great deal of work has been carried out on the preparation and application of ACC.

Since ACC is not a stable phase from the viewpoint of thermodynamics, temporal stabilization is achieved by the incorporation of organic molecules and specific ions in both biological and synthetic systems. The synthesis and stabilization of ACC have been pursued by freeze-drying,^12,13^ with polymers^14^ and proteins,^15,16^ or by using foreign ions like Mg^2+^,^17−19^ and phosphate.^20^ These approaches start from the Ca^2+^ and CO_3_^2–^ ion constituents in aqueous solution, and the crystallization process is stopped at the ACC stage by stabilizing the product kinetically.^21^

The local structure in ACC is a matter of debate.^22^ A “protostructuring” of ACC with respect to different crystalline CaCO_3_ polymorphs has been proposed,^23^ and the concept of “polyamorphism” has been considered for biogenic ACC.^22^ Although the short-range order in ACC is pH-dependent^24^ and OH^–^ groups were found to be incorporated in ACC,^25^ there are no indications for the presence of hydrogen carbonate (HCO_3_^–^) ions in ACC or any hydrated form of it.

Recent literature has shown that there is enormous potential for ACC precursor phases to be exploited in materials synthesis,^26^ with their promise of superior control over nucleation and growth and access to rapid growth rates and “non-crystalline” morphologies.^12−20^

ACC actually represents a family of phases whose structure and composition are dependent on the particular synthesis method and solution conditions (e.g., temperature, pH).^27^ Treatments following precipitation such as drying or washing with agents such as ethanol can also make significant changes to the ACC and its crystallization behavior.^28^ Consequently, synthetic ACC can vary considerably in terms of stability,^29^ coprecipitated ions, and the amount of structural and surface water.^30^ Upscaling of ACC synthesis has been studied as well.^31^

ACC can be used for several technological and industrial applications. Calcium carbonate cements were prepared from ACC that (re)crystallizes into calcite during the setting reaction. The hardened samples were microporous and showed excellent bioactivity rates, although their mechanical properties were poor.^32^ Three-dimensional objects were printed from long-term, Mg-stabilized ACC pastes with a high solid loading. This ACC remained stable for at least a couple of months, even after printing. Crystallization, if desired, occurred only after the 3D object was formed at low temperatures.^18^ Patterns of continuous 2D ACC films supported rat bone marrow stromal cell attachment and differentiation into osteoblast- and osteoclast-like cells.^33^ The aqueous instability of ACC, which leads to a fast release of its payload, was applied to realize burst drug release within cancer cells.^34^

Mechanochemistry ball-milling allows production of out-of-equilibrium structures, and it can be employed for breaking down bulk materials to nanosize.^35,36^ Defect formation on a large scale leads to an amorphization of crystalline solids.^37^ Ball milling treatment of CaCO_3_ has been studied.^38,39^ The transformation of calcite to aragonite has been reported,^40^ as well as the reverse transformation from aragonite to calcite.^41^ Similarly, vaterite was transformed to calcite mechanochemically.^42^ Recent studies have shown that ACC could be prepared by ball milling only using calcite, when Na_2_CO_3_ was used as a stabilizer of ACC.^43,44^

An important source of biogenic CaCO_3_ (bCC), which represents an alternative to the synthetic and geogenic ones, is given by waste seashells.^45,46^ These materials are formed from an ACC precursor and naturally contain organic macromolecules and ions that can stabilize ACC.^47^

The aim of this work is to compare the capability of different sources of CaCO_3_, from quarries or biogenic, to be converted into ACC. The main hypothesis is that the bCC can lead to a more stable ACC. The mollusk shells of oyster *C. gigas* (low-Mg calcite), scallop *P. jacobaeus* (medium-Mg calcite), and clam *C. gallina* (aragonite) were used as bCC sources. The working hypothesis is that different polymorphs, the content of Mg, and species-specific organic matrices can produce ACCs diverse in stability and transformation pathways into crystalline phases. The knowledge that can be gained from this research has a double interest for material science but also for a better understanding of the biomineralization processes. The novelty in this study is represented by the use of biogenic calcium carbonate, which was formed by ACC precursors before to stabilize in crystalline phases.

## Experimental Section

### Treatment of Samples

Seashells of *Crassostrea gigas*, *Pecten jacobaeus*, and *Chamelea gallina* were provided by F. Terzi (Palosco, BG, Italy). Samples were washed with tap water, treated with a 5 vol % sodium hypochlorite solution for 24 h to remove the organic residues from the seashell surface, washed again with deionized water, and air-dried. Then, the dry seashells were crushed by a hammer mill. gCC was provided by Italcementi spa. These powdered samples are referred to as starting samples. To achieve uniform particle size of the CaCO_3_ from the different sources, both geogenic and biogenic, the powders (30 g) were dry milled in a 500 mL zirconia jar together with 125 g of zirconia balls having a 20 mm diameter with the planetary ball milling PM 100 by Retsch (milling: 400 rpm, 1 h) and sieved at Ø < 45 μm. These samples are referred to as dry milled. For amorphization tests, 1.8 g of dry milled powder was wet milled in the zirconia jar together with 100 g of zirconia balls having a 2 mm diameter, 200 mL of solvent (ethanol, isopropanol, cyclohexane, heptane, or butanol), and 0.2 g of additive (Na_2_CO_3_, MgCO_3_, Li_2_CO_3_, K_2_CO_3_, and Ca(OH)_2_) and ground at 400 rpm in the planetary ball milling (milling: 400 rpm, pause: 20 min every 10 min of grinding), according to a reported procedure.^43^ Different grinding times, additives, and solvents were tested. These samples are termed wet milled.

### X-ray Powder Diffraction Analysis

X-ray powder diffraction (XRD) patterns were collected using a PanAnalytical X′Pert Pro diffractometer equipped with a multiarray X′Celerator detector using Cu Kα radiation (λ = 1.54056 Å), generated at 40 kV and 40 mA. The diffraction patterns were collected in the 2θ range between 20° and 60° with a step size (Δ2θ) of 0.02° and a counting time of 60 s. An estimation of the mass percentage of amorphous material, i.e., ACC, organic matter, and water, in the sample was calculated as the percentage of the integrated intensity of the amorphous diffraction band with respect to the integrated total diffraction intensity. We supposed that this diffraction band is due to the ACC, having a very low content of organic matrix and water and being materials that are formed by atoms with low diffraction scattering factors (i.e., carbon, nitrogen, oxygen, and hydrogen).

### Spectroscopic Analysis

A Thermo Scientific Nicolet iS10 FTIR Spectrometer was used to collect the FTIR spectra. The disk specimen for Fourier transform infrared (FTIR) analysis was obtained by mixing a small amount (about 2 mg) of the sample with 100 mg of KBr and applying a pressure of 45 psi (620.5 MPa) to the mixture using a press. The spectra were obtained with 4 cm^–1^ resolution and 64 scans.

### Thermogravimetric Analysis

Thermogravimetric analysis (TGA) was performed using an SDT Q600 V 8.0 instrument (TA Instruments). The system was pre-equilibrated at 30 °C, and then a ramp from 30 to 600 °C with a 10 °C min^–1^ heating rate was performed under nitrogen flow (100 mL min^–1^). The measurement was performed three times on 20 mg of each sample. The temperature range considered to estimate the content of the intraskeletal organic matter and water was between 150 and 450 °C.

### Particle Size Distribution Analysis

Particle size analyses were performed using a Malvern Mastersizer 2000 laser diffraction particle size analyzer (Malvern Panalytical). The particles from each sample were dispersed in 2-propanol for the measurement.

### Scanning Electron Microscopy Observations

The scanning electron microscopy (SEM) images were acquired using two different microscopes operating at 5 kV: a ZEISS Leo 1530 Gemini and a Thermo Fisher Quattro S equipped with a Schottky FEG. All samples were dried under vacuum in a desiccator, deposited on carbon tape, and 10 nm gold-coated before their observation.

### Transmission Electron Microscopy Investigations

The structure and composition analysis on the nanoscale was carried out with an FEI Tecnai F20ST high-resolution Transmission Electron Microscope (HR-TEM) operated at 200 kV. All samples were ground in an agate mortar and suspended in ethanol. The powders were deposited directly on a Cu grid with a holey-type carbon film. The solvent was evaporated at 50 °C for about 3 min.

### Inductively Coupled Plasma–Optical Emission Spectroscopy (ICP-OES)

All the powders (200 mg) were dissolved in HNO_3_ 50 vol % and were measured three times, 12 s each, with 60 s of prerunning, using ICP-OES, Spectro Arcos-Ametek, Inductive Coupled Plasma–Optical Emission Spectroscopy with an axial torch, and high salinity kit. The calibration curve was made using certified standards in water.

## Results and Discussion

### Synthesis and Characterization of the Starting Materials

As a source of biogenic CaCO_3_ (bCC) particles, this research used seashells from species that have (i) a strong relevance in aquaculture, (ii) are made of a single CaCO_3_ polymorph, and (iii) have diverse crystalline textures. They were the oyster *C. gigas*, the scallop *P. jacobaeus*, and the clam *C. gallina*, the shells of which were made of low-Mg calcite (about 1 mol %), medium-Mg calcite (1.4 mol %), and aragonite, respectively. Control experiments were performed using gCC, made of calcite. The elemental, compositional, and structural characterization of these materials is reported in Table SI1.

The starting materials were dry-milled and sieved to homogenize the particle size. The obtained materials, termed dry milled, were used for the subsequent amorphization experiments. The compositional and structural features of these materials are reported in Table 1 and Figure SI1.

**Table 1:** Percentage of CaCO_3_ Polymorphs, Organic Matrix Content, Grain Size, Surface Area, and Crystallite Size of gCC, Oyster Shell, Scallop Shell, and Clam Shell Powder after Dry Ball Milling and Sieving at Ø < 45 μm with Instrumental Error Reported

Sample	Calcite (wt %)^a^	Aragonite (wt %)^a^	ACC (wt %)^b^	O.M. (wt %)	D_50_ (μm)	S. A. (m^2^/g)	d_(104)_/d_(111)_^c^ (nm)
geo CaCO_3_	100 ± 2	–	0 ± 2	0 ± 0.1	8.34	4.3	14.4 ± 0.2
oyster shell	97 ± 2	–	3 ± 2	0.7 ± 0.1	8.13	7.6	13.9 ± 0.2
scallop shell	90 ± 2	4 ± 2	6 ± 2	0.9 ± 0.1	15.54	6.6	13.7 ± 0.2
clam shell	7 ± 2	89 ± 2	4 ± 2	0.6 ± 0.1	13.80	7.3	15.8 ± 0.2/12.3 ± 0.3

a Percentage of crystalline phases.

b Percentage of ACC in the particles.

c The crystallite size was calculated along the (104) and the (111) zone axis for calcite and aragonite, respectively. O.M. indicates intraskeletal organic matter and water. D_50_ indicates that up to 50% percent of the total particles have a grain size smaller than the reported value. S.A. indicates specific surface area. d_(hkl)_ indicates the size of the crystalline domain along the direction indicated by the Miller indices.

The diffraction patterns (Figure SI1, Table 1) showed that a transition from aragonite to calcite associated with the dry grinding process occurred in clam shell powder, an effect already reported in the literature.^48^ The calcite phases were not affected by the grinding process.

The analyses of the XRD diffraction patterns showed that gCC particles are free of ACC, while the bCC particles contain ACC in a percentage that is species-dependent. The presence of ACC is expected since this phase is a precursor of the crystalline phases in the formation of mollusk shells and, in general, of many calcifying organisms. The content of ACC reported in Table 1 has been evaluated by the X-ray diffraction patterns. It also includes contributions from the intraskeletal organic matter and water. It is considered that these latter components have a minimal effect on the overall scattering, being composed of elements like carbon, nitrogen, oxygen, and hydrogen that have a low X-ray scattering factor with respect to calcium. The content of water, which could increase the intensity of the amorphous diffraction band, is below 1 wt %, as indicated by the TGA data that always show a content of water and organic matter lower than 1 wt % (Table 1; Figure SI2). This content is very low in the gCC values, as expected.

The elemental composition agrees with the literature data,^49^ with calcite and aragonite able to host Mg ions and Sr ions in the crystalline lattices, respectively.

**Figure 1:**
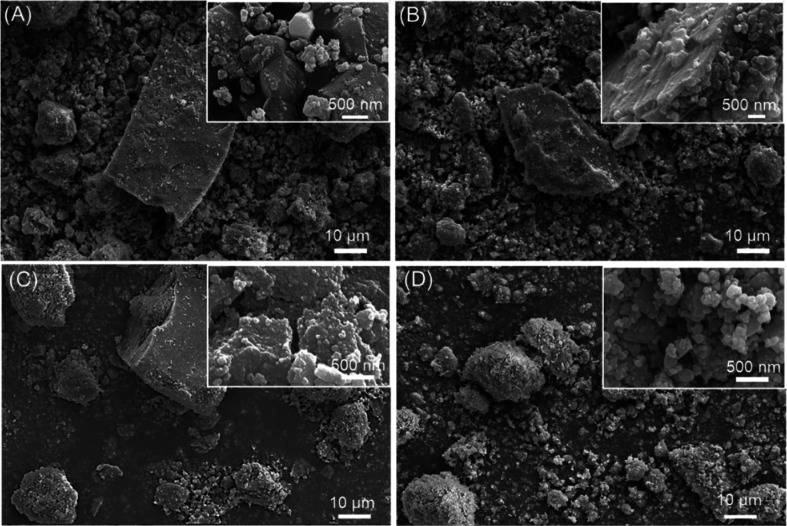
SEM images of (A) gCC, (B) oyster shell, (C) scallop shell, and (D) clam shell powders dry milled. The inset shows a higher-magnification view.

In order to study the morphology of the dry-milled powders, SEM images were collected (Figure 1). The gCC powder appeared as a mixture of big particles (around 35 μm) and small ones of about 1 μm size. Many of them were showing typical crystalline shapes in which rhombohedral {104} faces are visible in the small grain. The big particles are aggregates in which the crystalline faces are not clearly defined. The powder from oyster shells was also heterogeneous, but in this case, the bigger particles were preserving the typical foliated structure of the magnesium calcite shells, and the small ones appeared as spheroidal grains. Their size along the main axis was about 500 nm. The powder from the scallop shell was similar to that from the oyster shell, with the difference that the big particles did not show the foliated crystalline texture. A different scenario was shown by the powder from the clam shells. In this case, the bigger particles appeared as aggregates of the small ones, which were spheroids that had a size lower than 500 nm along their main axis. The morphological analysis of the data agrees with the measurements of the specific surface area. The bCC powders, having a family of smaller particles with smaller sizes than the geogenic ones, have a higher surface area (6–7 m^2^ g^–1^) compared to the gCC (about 4 m^2^ g^–1^). The dimension of the crystalline domains does not show a correlation with the above experimental data.

#### Optimization of the CaCO_3_ Mechanochemical Process

This amorphization process of CaCO_3_ finds inspiration from the work of Opitz et al.^43,44^ The optimization of the experimental conditions was performed by using bCC from oyster shells. The optimal ball milling time was evaluated using 10 wt % Na_2_CO_3_ as an additive and cyclohexane as the dispersion liquid. Ball milling times of 1, 3, 6, 12, and 24 h were used (Figure SI3). The analyses of the diffraction patterns indicated that after 6 h of ball milling, the highest content of ACC was observed from bCC (Table 2, Figure SI3). Fixing the ball milling time at 6 h, the effect of several solvents such as ethanol, isopropanol, cyclohexane, heptane, and butanol was tested, keeping 10 wt % Na_2_CO_3_ as the additive (Figure SI4). A higher amorphization degree was obtained using cyclohexane, suggesting that the lower the solubility of water in the solvent, the higher the degree of amorphization (Figure SI4). Such an observation could mean that by displacing water, the recrystallization of ACC is prevented. Indeed, it has been reported that increased water content accelerates the transformation of ACC in crystalline phases.^50^

**Table 2:** Percentage of CaCO_3_ Polymorphs, Organic Material Content, and Crystallite Size of gCC, Oyster Shell, Scallop Shell, and Clam Shell Powder after Different Times of Ball Milling Using Cyclohexane As Dispersing Agent and 10 wt % Na_2_CO_3_ as Additive for Amorphization with the Instrumental Error Reported

Sample	Mill time (hour)	Calcite (wt %)	Aragonite (wt %)^a^	ACC (wt %)^b^	O.M.^b^ (wt %)	d_(104)_/d_(111)_^c^ (nm)
geo CaCO_3_	1	70 ± 2	–	30 ± 2	0.1 ± 0.1	11.1 ± 0.3
	6	64 ± 2	–	36 ± 2	0.1 ± 0.1	8.3 ± 0.1
	24	58 ± 2	–	42 ± 2	0.1 ± 0.1	7.5 ± 0.1
oyster shell	1	79 ± 2	5 ± 2	16 ± 2	0.5 ± 0.1	14.2 ± 0.5
	6	42 ± 2	18 ± 2	40 ± 2	0.5 ± 0.1	6.7 ± 0.2/12.0 ± 1.2
	24	45 ± 2	26 ± 2	28 ± 2	0.5 ± 0.1	7.1 ± 0.2/13.7 ± 0.9
scallop shell	1	65 ± 2	10 ± 2	25 ± 2	0.4 ± 0.1	14.3 ± 0.6/15.0 ± 1.0
	6	38 ± 2	16 ± 2	46 ± 2	0.5 ± 0.1	8.9 ± 0.3/12.2 ± 0.8
	24	27 ± 2	68 ± 2	5 ± 2	0.6 ± 0.1	29.0 ± 3.0/40.0 ± 3.0
clam shell	1	11 ± 2	68 ± 2	21 ± 2	0.7 ± 0.1	11.5 ± 0.7/18.6 ± 0.3
	6	29 ± 2	31 ± 2	39 ± 2	0.8 ± 0.1	6.7 ± 0.1/12.0 ± 0.3
	24	72 ± 2	23 ± 2	4 ± 2	0.7 ± 0.1	24.0 ± 1.0/52.0 ± 22.0

a Percentage of crystalline phases.

b Percentage of ACC in the powder material determined by analyzing the X-ray diffraction profile. O.M. indicates intraskeletal organic matter and water.

c The crystallite size was calculated along the (104) and the (111) zone axis for calcite and aragonite, respectively.

Having optimized ball milling time and solvent, trials were performed using different additives, such as Na_2_CO_3_, MgCO_3_, Li_2_CO_3_, K_2_CO_3_, and Ca(OH)_2_ (Figure SI5). The material obtained using Na_2_CO_3_ as an additive showed a higher amorphization degree. Interestingly, when MgCO_3_ was used as an additive, Mg substitution in the calcite phase occurred (Figure SI5).

### Reduction of Crystalline Domain Size and Amorphization of CaCO_3_ from Different Sources in the Presence of Na_2_CO_3_

**Figure 2:**
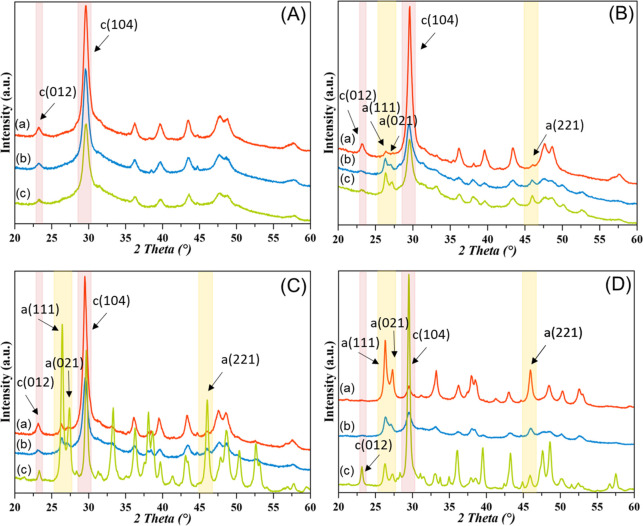
Powder X-ray diffraction patterns of (A) gCC, (B) oyster, (C) scallop, and (D) clam shell powders wet milled for (a) 1, (b) 6, and (c) 24 h. The diffraction patterns were indexed accordingly to the PDF 00–005–0586 for calcite and PDF 00–005–0453 for aragonite. The intensities are in linear scale. The X-ray diffraction patterns of the starting materials (0 h) are reported in the Supporting Information (Figure SI1).

Mechanochemical experiments on gCC and bCC from the three mollusk species were performed using cyclohexane as a solvent and 10 wt % of Na_2_CO_3_ as an amorphizing additive. The powders milled for 1, 6, and 24 h were characterized by several techniques that provided converging results (Figures 2 and SI6; Table 2). A complete amorphization was never achieved; this observation contrasts with the experiments of Tremel et al.^43^

However, it has to be considered that in the presented research different experimental conditions were used (e.g., a bigger jar, 500 mL versus 10 mL) with the aim to also test experimental conditions relevant for a process scale-up. Among the bCC, the one formed by Mg-calcite showed a higher degree of amorphization, being 40 and 49 wt % for oyster and scallop powder, respectively. Under the same experimental conditions, the clam powder amorphized for 39 wt %. These degrees of amorphization were achieved after 6 h of grinding. Interestingly, the gCC had a degree of amorphization of 36 wt % after 6 h of ball milling, and this value increased to 42 wt % after 24 h of ball milling. Diversely, the degree of amorphization in bCC for times longer than 6 h decreased, and the conversion into a different polymorph occurred. The oyster and the scallop powders converted to aragonite, and this occurred more for scallops than oysters.

The higher content of Mg ions in scallops can justify this observation since Mg ions kinetically favor the formation of aragonite,^1^ but since the solvent used is almost water-free, this classical explanation supported by the strong hydration sphere of Mg ions cannot be applied. On the contrary, clam powder converted to calcite upon longer time of ball milling. The conversion of aragonite to calcite has been reported as a solid phase transition,^51^ but this mechanism could not occur during the milling process in cyclohexane in the presence of Na_2_CO_3_. The diffraction peaks broadening process should be due to a reduction of the crystalline domain sizes with the ball milling time, which may proceed with the generation of defects in the crystalline domain during the plastic deformation.^52^ Accordingly, the bCC powders show shorter crystalline domain sizes after 6 h of grinding (Table 2).

The experimental data indicated that in the absence of Na_2_CO_3_, the formation of the ACC does not occur, and only a reduction of the crystalline domain size is observed. The role of the Na_2_CO_3_ in stabilizing ACC was demonstrated by Leukel et al.,^43^ who suggested that Na ions stabilize ACC. The proposed mechanism reports that the formation of ACC from calcite occurs due to the high energy released during the ball milling process. It can be controversial to demonstrate the reason for which the stable phase of calcite transforms to the unstable solid phase ACC. We may suppose that the transformation of a crystalline CaCO_3_ to ACC may take place via a surface liquid state, which may form when Na_2_CO_3_, or similar substances, generates relatively low eutectic temperature composites,^53,54^ when in contact with solid CaCO_3_ at elevated temperatures due to the friction in grinding.^55^ This potential melted doped CaCO_3_ may rapidly be cooled and may transform to ACC or other phases.

**Figure 3:**
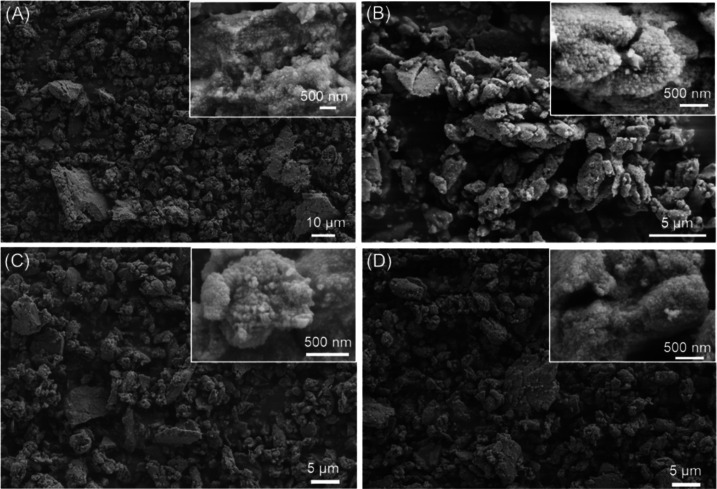
SEM images of (A) gCC, (B) oyster shell, (C) scallop shell, and (D) clam shell powders wet milled for 6 h. The inset shows a higher-magnification view.

The morphology of the powders obtained after 6 h of ball milling, the condition that produced the higher content of ACC from bCC, was investigated by SEM (Figure 3). The samples, when observed at low magnifications, appeared as aggregates having wide distributions in size and assuming diverse shapes. The high-magnification images showed that the aggregates were formed of nanoparticles having a size below 500 nm. Interestingly, the gCC nanoparticles were more compact than the bCC ones. This differentiation could be associated with a higher surface stabilization of the bCC nanoparticles from the various components, molecules, and ions of the pristine shells. The geogenic ones have a high surface energy missing the biological stabilization and strongly aggregate in compact big particles. This consideration is supported by the estimation of the content of the intracrystalline organic matrix, a unique signature of the bCC, that remains almost constant during the grinding processes, as reported in Table 2.

**Figure 4:**
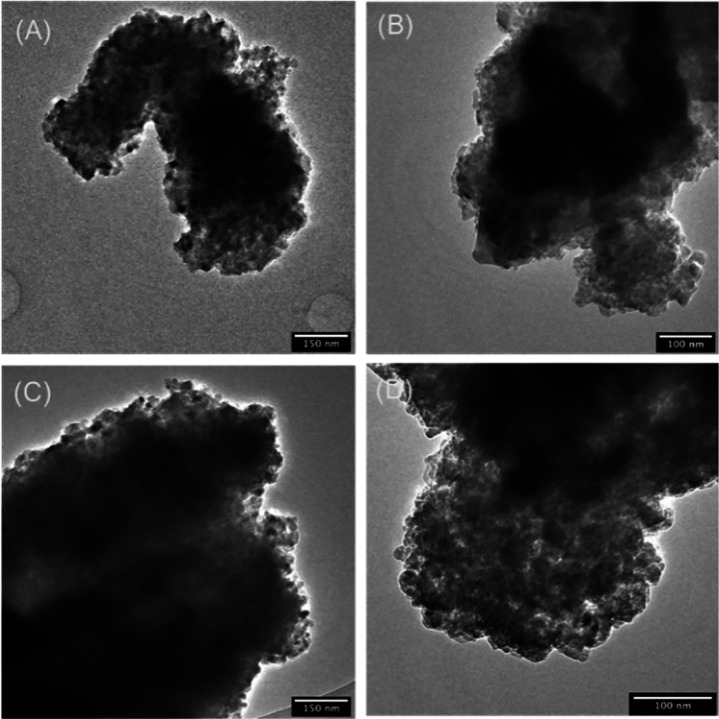
TEM images at low magnification of (A) gCC, (B) oyster shell, (C) scallop, and (D) clam powders wet ball milled for 6 h.

The mechanochemically treated CaCO_3_ materials were also investigated by transmission electron microscopy (TEM) and selected area electron diffraction (SAED). The low-magnification TEM images (Figure 4) showed that the general morphology of all samples was represented by aggregates formed by the aggregation of smaller structures with different dimensions and, in some regions, quite regular shapes. This suggested the copresence of domains with a certain degree of crystallinity and amorphous domains, which were randomly superimposed on each other.

**Figure 5:**
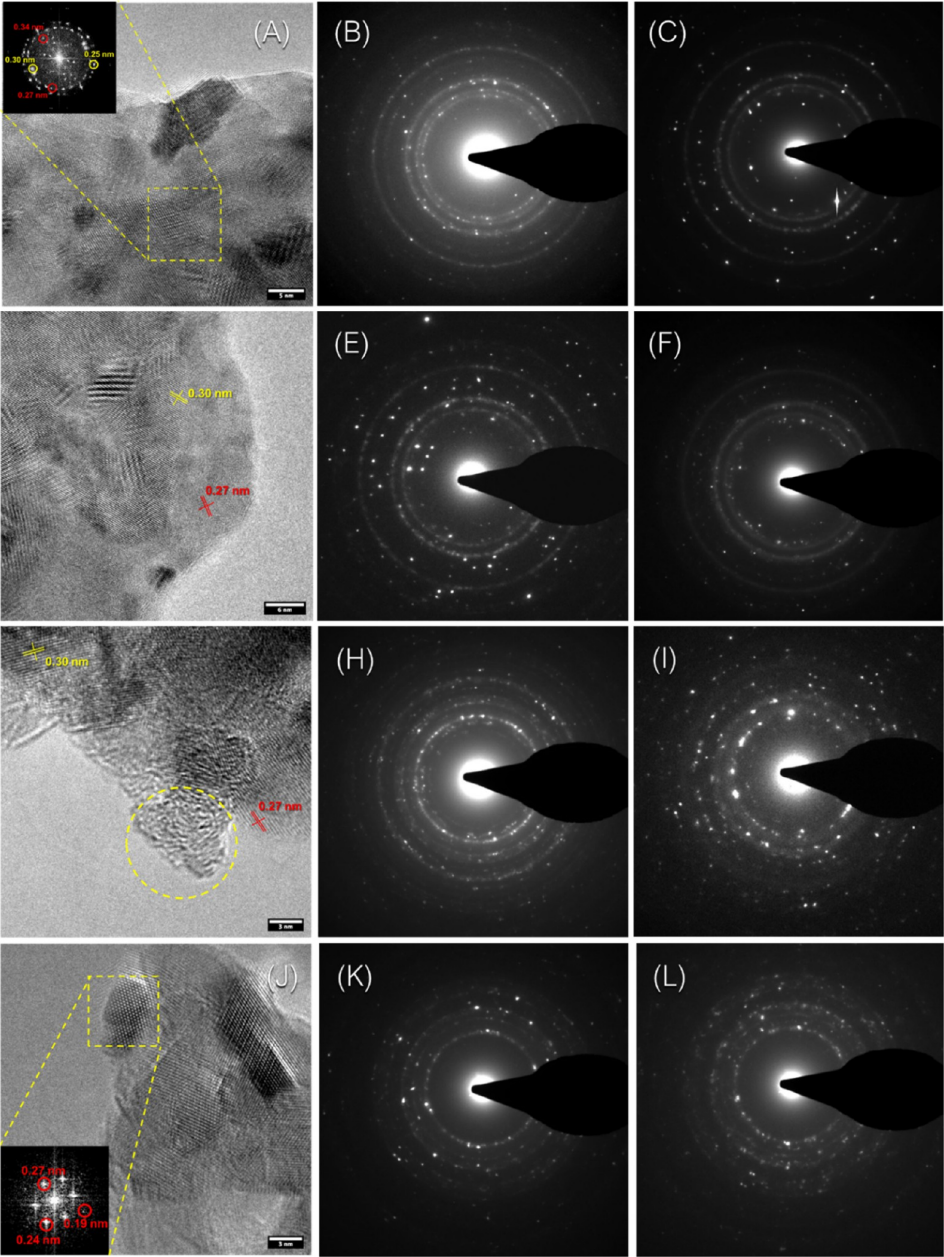
High-Resolution TEM and Selected Area Electron Diffraction micrographs of (A–C) gCC, (D–E) oyster shell, (G–I) scallop, and (K–L) clam powders wet milled for 6 h. The inset reports the Fourier transform of a crystalline region, indicated by the yellow square. The yellow circles show an amorphous region. The selected area election diffraction micrographs were collected in different regions of the specimen, and the two most representative ones are reported.

Additional information about the structure of the mechanochemically treated material was obtained from the high-resolution TEM (HRTEM) images and the SAED patterns reported in Figure 5. The geogenic calcite sample (Figure 5A) produced HRTEM images in which the interplanar distances of calcite and, in a few cases, aragonite were detected. They could be easily observed through the Fourier transform in the inset (Figure 5A). The SAED patterns collected by centering the aperture on two different aggregates, to ensure a better representation of the sample, confirmed the main presence of calcite. The patterns were typical for a polycrystalline structure, due to the aggregation of crystallites of different dimensions and orientations. The evaluated typical *d*-spacings of calcite are reported in Table SI2. In Figure 5C, the presence of ACC was not directly observed as single particles from the HRTEM images, suggesting that this phase was embedded in the aggregate particles. Thus, the observation of ACC domains was hindered by the coexistence with crystalline domains.

A quite similar scenario was observed when the mechanochemically treated bCC samples were analyzed (Figure 5D–L). The HRTEM images from the oyster and scallop powders show a qualitative presence of more intense diffraction peaks for calcite, while in the clam shells, the HRTEM images show the typical and more intense spot of aragonite, as illustrated in the Fourier transform insets. These results perfectly match with SAED patterns that display a diffraction spot structure typical of a polycrystalline structure, due to the aggregation of many crystallites of different dimensions and orientations. As discussed for the geogenic calcite, the presence of ACC is not very evident.

We suggest that also for the bCC, the ACC coexists with the crystalline phases, and the amorphization process occurs by a possible solid-state transformation involving the progressive reduction of the dimension of the crystalline domains, as also suggested by the analysis of the powder XRD data. Such a phenomenon has been reported for geogenic calcite:^52^ the researchers showed that mechanical milling was effective in reducing the domain size to the nanoscale and introducing large microstrains. They suggested that the milling process mostly involves impulsive forces between the vial, grinding bodies, and powder. Even if the average temperature can be kept constant, the energy released locally during the fast impacts is considerably high. Under these conditions, it could be put forward that dislocation glide should be favored with respect to twinning as the active plasticity mechanism.^52^

#### Time Stability of ACC According to Source and Aging Environment

**Figure 6:**
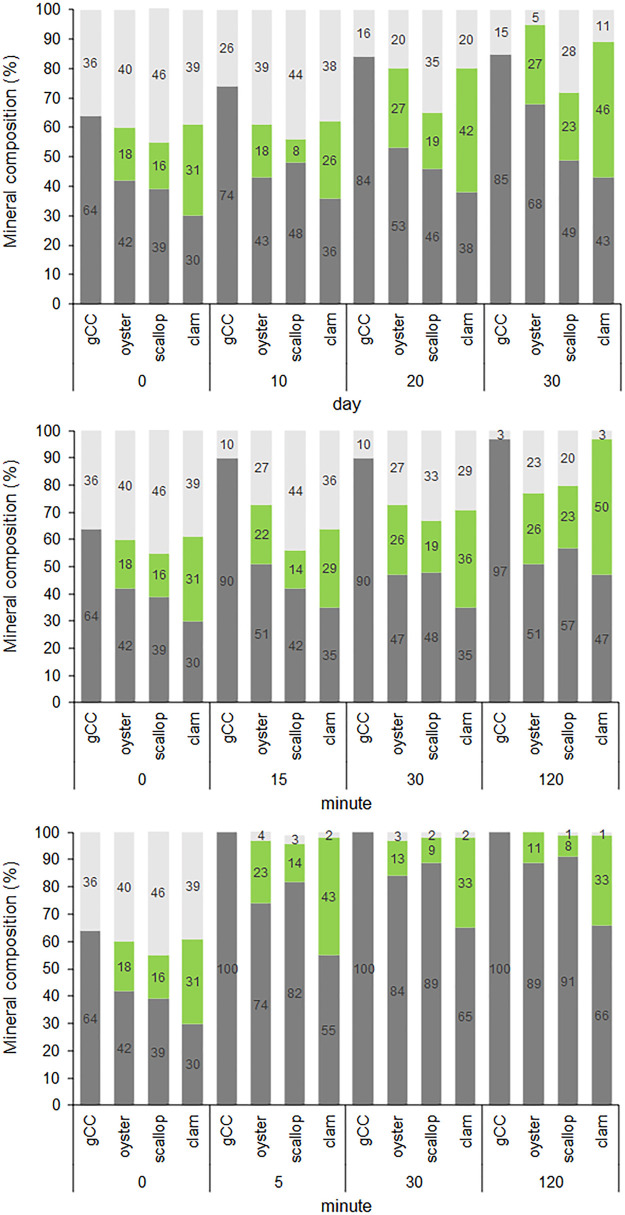
Mass percentage of CaCO_3_ polymorphs (light grey: ACC; green: aragonite; dark grey: calcite) of gCC, oyster shell, scallop shell, and clam shell powder after different aging times in diverse environments: (A) water; (B) ethanol; (C) nitrogen.

**Figure 7:**
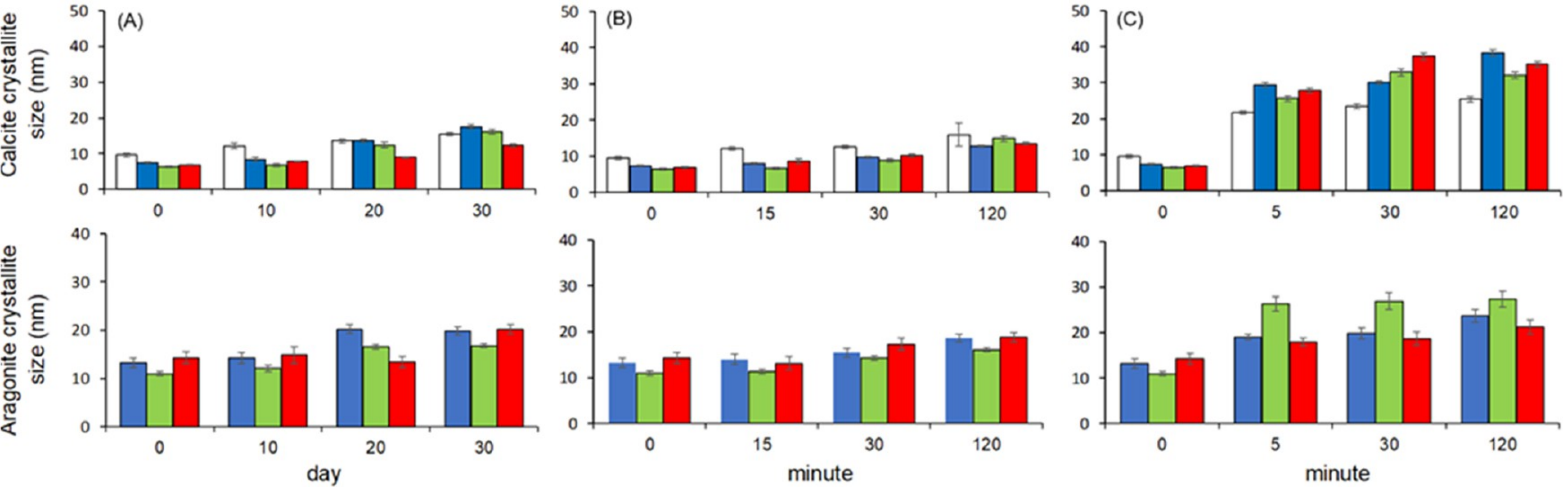
Histograms of calcite and aragonite crystallite size of gCC (white), oyster shell (blue), scallop shell (red), and clam shell (green) powder after different aging times in diverse environments: (A) nitrogen, (B) ethanol, and (C) water. The error bars indicate the standard deviation.

To study the effects of aging time on the recrystallization process of ACC and the increase in the size of the crystalline domains, the powders wet milled for 6 h were stored in different environments: (i) in a nitrogen atmosphere for 10, 20, and 30 days; (ii) in ethanol under bar-stirring for 15, 30, and 120 min; (iii) in H_2_O under bar-stirring for 5, 30, and 120 min. The results are illustrated in Figures 6 and 7 and reported in Table SI3.

In all of the investigated environments, the gCC converted ACC into calcite. This process was very slow in a nitrogen atmosphere; even after one month, the transition from ACC to calcite was not complete (Table SI3; Figure 6). On the contrary, in ethanol and water, the conversion was complete and occurred in 2 h and 5 min, respectively (Table SI3; Figure 6). The aging was also associated with an increase of the crystalline size, which was of similar extent in nitrogen or ethanol environments after the longest time of aging but higher in water (Table SI3; Figure 7). This suggests that in water, the recrystallization process is more efficient, as expected, considering the higher solubility of CaCO_3_ in this solvent with respect to ethanol. Thus, it is supposed that a dissolution reprecipitation process occurs in the transformation of ACC in the crystalline phase, as supposed above considering the ACC in different water containing solvents. This mechanism of transformation of ACC in aqueous systems has already been reported.^50,56^

The aging of bCC particles gave different results with respect to gCC. The powder from oyster shells, when located in a nitrogen or ethanol environment, over time increased the relative content of calcite while the content of aragonite was almost constant, while it decreased in water (Table SI3; Figure 6). This last effect can be also associated with the highest solubility of CaCO_3_ in water with respect to ethanol.^57^ In all the aging experiments, the size of the crystalline domains of calcite increased, having the most marked increase in water (Table SI3; Figure 7). A similar trend was observed for the size of the crystalline domains of aragonite (Table SI3; Figure 7).

When the powder from scallops was aged, a behavior different from that of oysters was observed. In this case, with the aging, both the relative amount of calcite and aragonite increased, while the relative content of ACC was progressively decreasing (Table SI2; Figure 6).

The powder from the clam aged in nitrogen and ethanol environments results in a relevant increase of the relative content of aragonite and calcite, while in water over time, the relative content of aragonite decreased, suggesting a transition from aragonite to calcite (Table SI2; Figure 6). The size of the crystalline domains increased with time for both calcite and aragonite, irrespective of the environment in which the powders were located (Table SI3; Figure 7).

The above data indicated, as expected, that the major stability of the ACC occurred in a nitrogen environment. Indeed, the particles in this gas remain quite stable, and only solid-state transition can occur, diversely from the ethanol or water environments, where CaCO_3_ dissolution and reprecipitation processes can take place.

**Figure 8:**
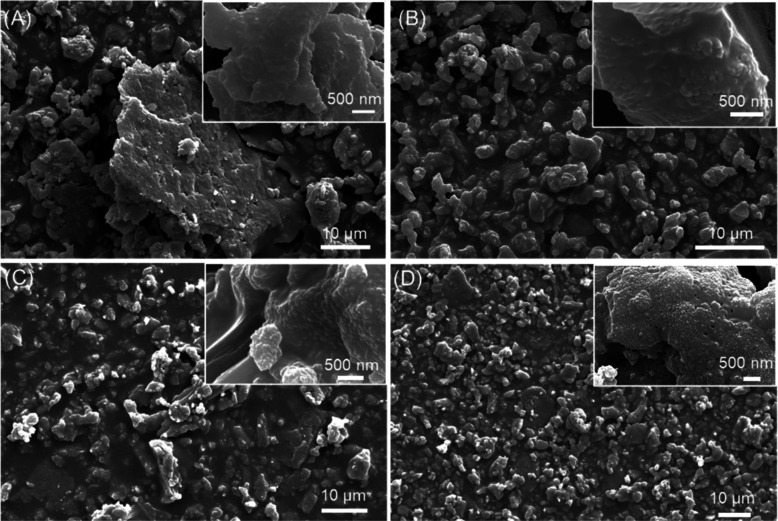
SEM images of the (A) gCC, (B) oyster shell, (C) scallop shell, and (D) clam shell powders wet milled for 6 h and recrystallized after 3 months in N_2_ environment. The inset shows a higher-magnification view.

The samples aged for three months in a nitrogen environment were investigated by SEM to detect whether some solid-state morphological reorganization occurred. The images reported in Figure 8 indicated that the grains aggregated in bigger particles have different textures, with the most crystalline one observed for the clam powder.

## Conclusions

Ball milling mechanochemistry treatments on bCC and gCC have shown that the amorphization process occurred via a progressive reduction of the sizes of the crystalline domains and may suggest a mechanism that involves the formation of a low-temperature eutectic liquid phase in the presence of Na_2_CO_3_. For all the samples, although to a diverse extent, ACC coexisted with crystalline domains. For the bCC, the conversion of the crystalline phase in ACC occurred with the concomitant transition of calcite to aragonite and vice versa.

On the contrary, gCC converted only into ACC. In addition to this, when increasing the ball milling time, the ACC from bCC converted into crystalline phases, while gCC increased the content of ACC and decreased the size of the crystalline domains. This diverse behavior could be attributed to the unique presence in the bCC of the intraskeletal organic matrix and biologically relevant trace elements. The phase transformation of CaCO_3_ to ACC may occur via a surface eutectic liquid state, which may occur in the presence of Na_2_CO_3_, that then may transform to ACC or crystalline phases.

The aging in different environments, nitrogen, ethanol, or water, of mechanochemically treated bCC and gCC produced different results, the former being converted into aragonite and calcite and the latter only into calcite.

In view of the potential application of ACC, this research showed that the use of bCC offers a wider scenario of structural features and stabilities with respect to the gCC. This is particularly relevant when different materials are required for diverse applications.

## Supporting Information Available

TGA profiles, FTIR profiles, XRD patterns, SAED patterns analysis (PDF)
