# Supplementary material for: Nanocrystalline and Amorphous Calcium Carbonate from Waste Seashells by Ball Milling Mechanochemistry Processes
Source: Cryst Growth Des. 2023 Dec 22;24(2):657–68. doi: 10.1021/acs.cgd.3c01007 (PMC10797593; doi:10.1021/acs.cgd.3c01007)
Supplement: Supplementary file 1 — cg3c01007_si_001.pdf [file cg3c01007_si_001.pdf]

Supporting Information

Nano-crystalline and amorphous calcium carbonate from waste seashells by ball milling mechanochemistry processes

Chiara Marchini,[1] Carla Triunfo,[1,2] Nicolas Greggio,[3] Simona Fermani,[1] Devis Montroni,[1] Andrea Migliori,[4] Alessandro Gradone,[4] Stefano Goffredo,[2,3] Gabriele Maoloni,[5] Jaime Gómez Morales,[6] Helmut Cölfen,[7] and Giuseppe Falini[1,]*

[1] Department of Chemistry "Giacomo Ciamician", University of Bologna, via F. Selmi 2, 40126 Bologna, Italy, *email: giuseppe.falini@unibo.it*. [2] Fano Marine Center, viale Adriatico 1/N 61032 Fano, Italy. [3] Department of Biological, Geological and Environmental Sciences, University of Bologna, via F. Selmi 3, 40126 Bologna, Italy. [4] Institute for Microelectronics and Microsystems (IMM) - CNR section of Bologna, via P. Gobetti 101, 40129 Bologna,Italy. [5] Finproject S.p.A., Plant Ascoli Piceno, Via Enrico Mattei, 1-Zona Ind.le Campolungo, 3100 Ascoli Piceno, Italy. [6] Laboratorio de Estudios Cristalográficos, Instituto Andaluz de Ciencias de la Tierra (CSIC-UGR), Avda Las Palmeras 4, 18100 Armilla (Granada), Spain. [7] Department of Chemistry, Physical Chemistry, University of Konstanz, Universitätsstrasse 10, Box 714, D-78457 Konstanz, Germany.

Figure SI1 pag. SI2

Figure SI2 pag. SI2

Figure SI3 pag. SI3

Figure SI4 pag. SI3

Figure SI5 pag. SI4

Figure SI6 pag. SI4

Table SI1 pag. SI5

Table SI2 pag. SI5

Table SI3 pag. SI6

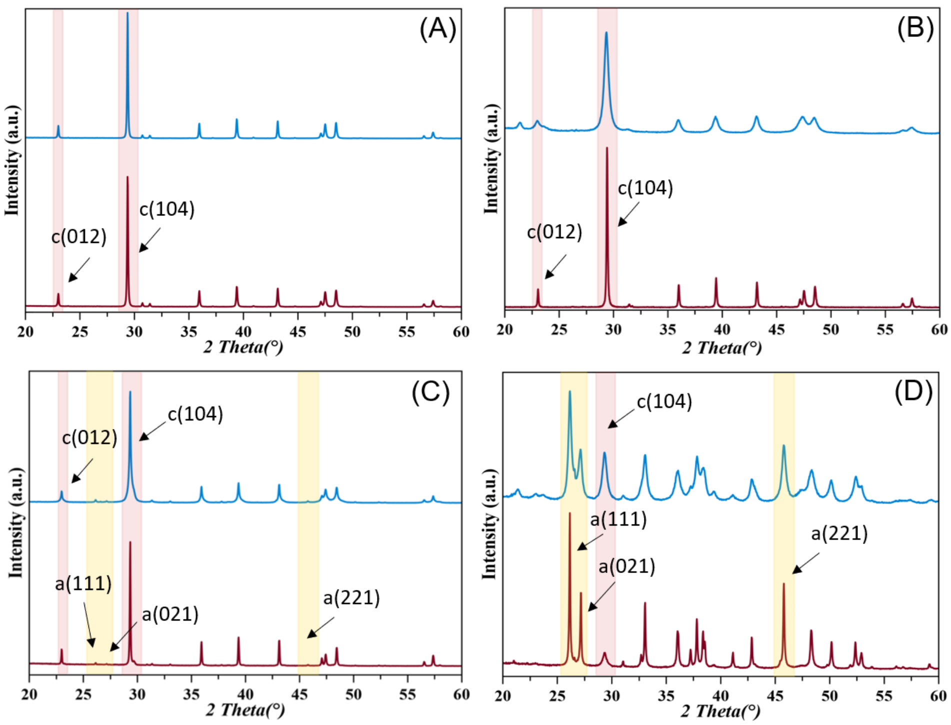

Figure SI1. X-ray powder diffraction patterns from starting materials (red line) and dry milled materials (blu line) of geogenic $CaCO_3$ (A), oyster shells (B), scallop shells (C), and clam shells (D). The intensity is in linear scale. The diffraction patterns were indexed according to PDF 00-005-0586 and PDF 00-005-0453 for calcite and aragonite, respectively.

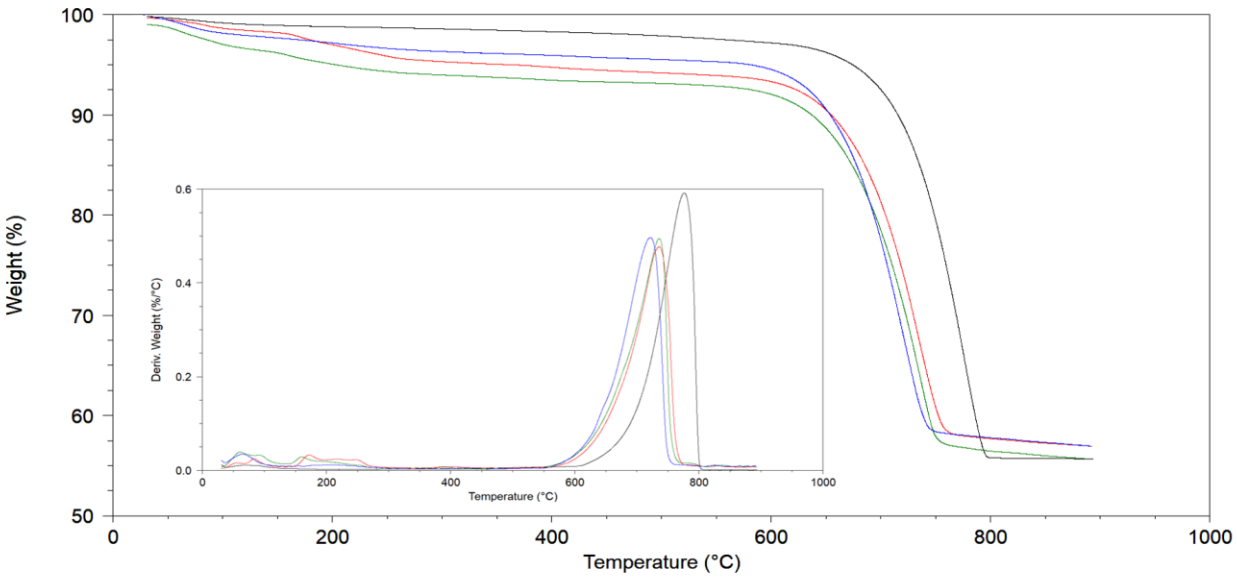

Figure SI2. Thermogravimetric analysis (TGA) profiles of geogenic $CaCO_3$ (black), oyster (blu), scallop (red), and clam (green). The temperature range considered to estimate the content of intraskeletal organic matrix was between 300 °C and 500 °C.

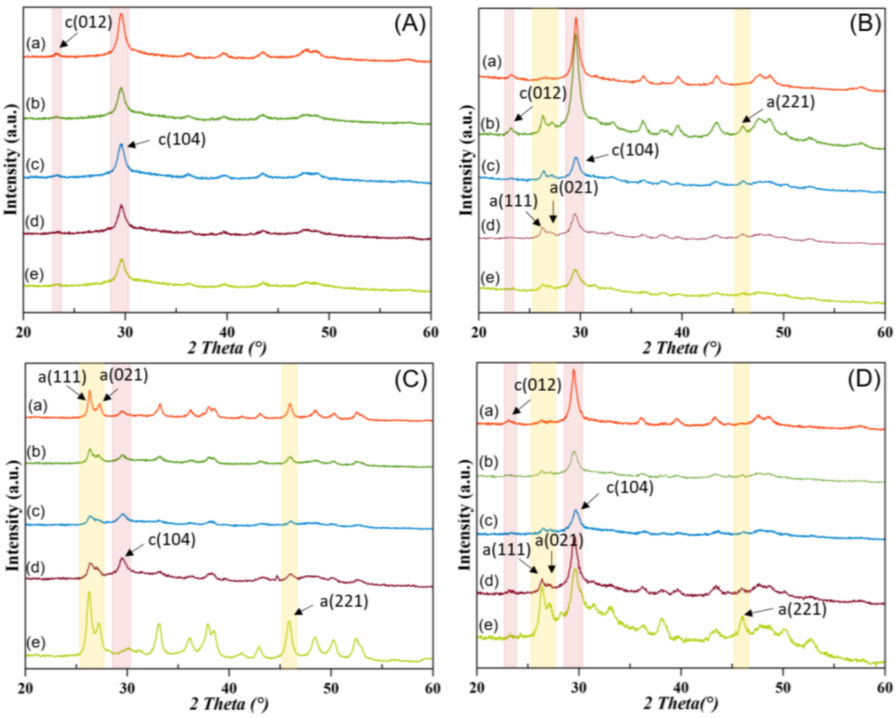

Figure SI3. Powder X-ray diffraction patterns of geogenic $CaCO_3$ (A), oyster (B), scallop (C), and clam (D) shells wet milled for 1 hour (a), 3 hours (b), 6 hours (c), 12 hours (d), and 24 hours (e). The intensity is in linear scale. The diffraction patterns were indexed according to PDF 00-005-0586 for calcite and PDF 00-005-0453 for aragonite.

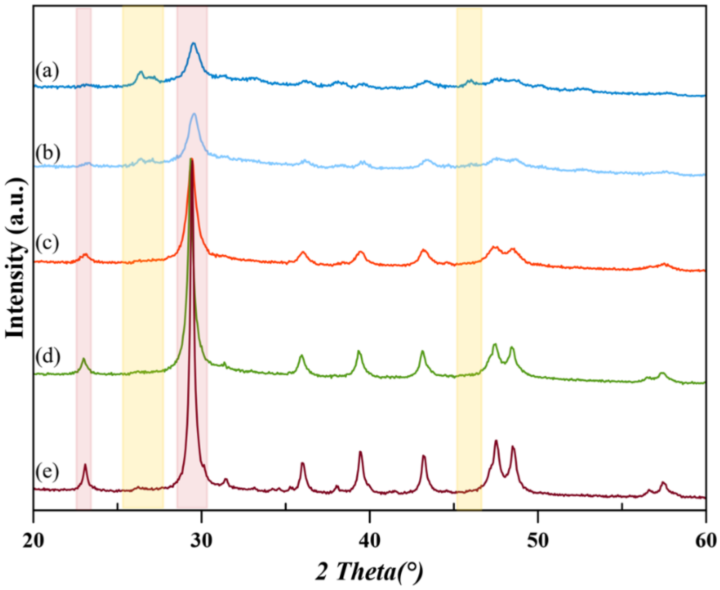

Figure SI4. Powder X-ray diffraction patterns of oyster wet milled for 6 hours with different solvents: a) Cyclohexane; b) Heptane; c) Isopropanol; d) Ethanol; e) Butane. The intensity is in linear scale. The diffraction patterns were indexed according to PDF 00-005-0586 for calcite and PDF 00-005-0453 for aragonite.

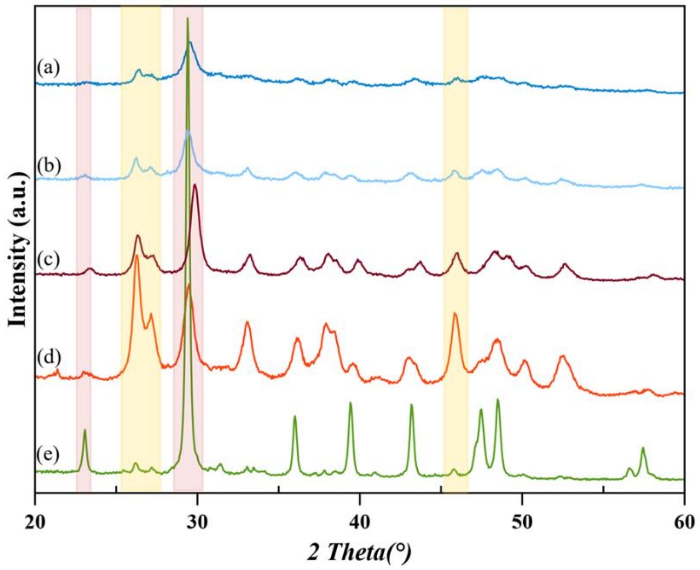

Figure SI5. Powder X-ray diffraction patterns of oyster wet milled for 6 hours with different additives: a) Na_2CO_3; b) $Ca(OH)_2$; c) $MgCO_3$; d) Li_2CO_3; e) K_2CO_3. The intensity is in linear scale. The diffraction patterns were indexed according to PDF 00-005-0586 for calcite and PDF 00-005-0453 for aragonite.

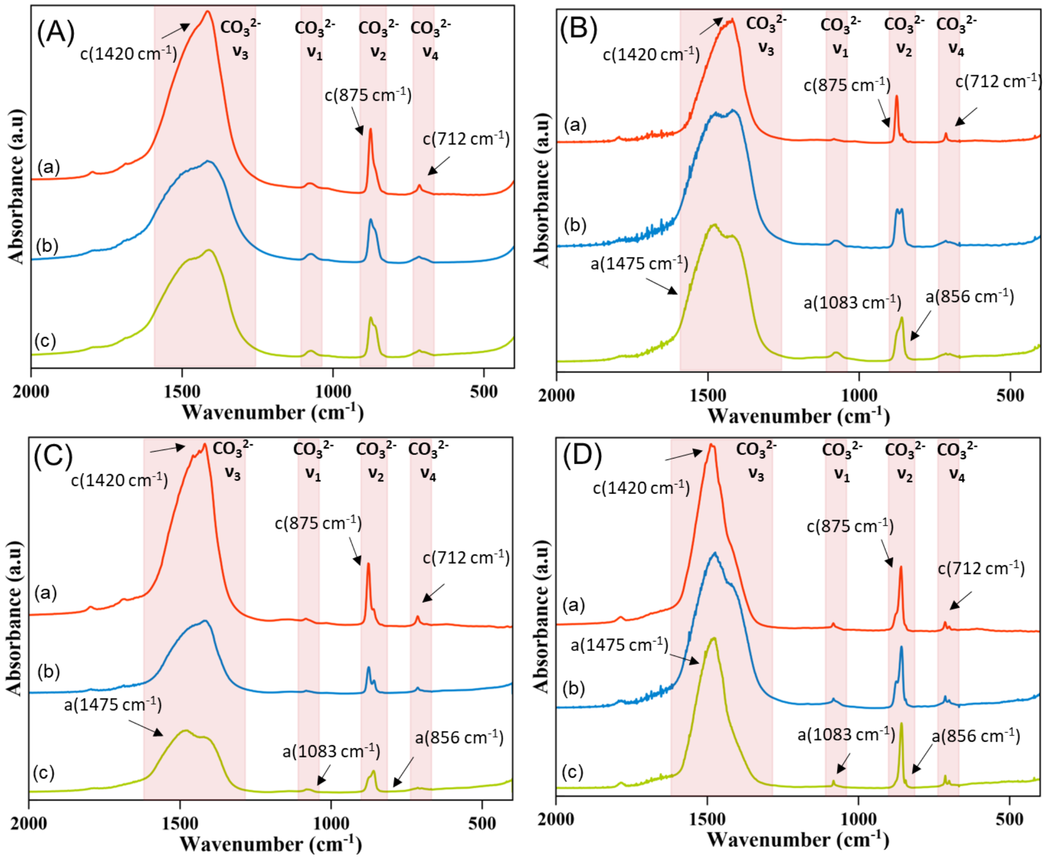

Figure SI6. FTIR spectra of geogenic $CaCO_3$ (A), oyster (B), scallop (C), and clam (D) shells wet milled for 1 hour (a), 6 hours (b), and 24 hours (c).

Table SI1. Percentage of $CaCO_3$ polymorph, organic matrix content, elemental composition, and crystallite size of geogenic calcium carbonate, oyster shell, scallop shell and clam shell powder after hammer mill grinding. The crystallite size was calculated along the (104) and the (111) zone axis for calcite and aragonite respectively.

**Sample**	**Calcite** (wt. %)*	**Aragonite** (wt. %)*	**Organic material content** (wt. %)[#]	**Mg** (at. %)	**Sr** (at. %)	$\mathbf{d_{(104)}/d_{(111)}}$ (nm)
geo $CaCO_3$	100	/	0.2	1.24	0.00	1525 ± 3/ -
oyster shell	99.41	0.59	1.1	0.99	0.09	92 ± 3/ -
scallop shell	96.23	3.77	0.7	1.36	0.04	88 ± 3/ 70 ± 16
clam shell	5.78	94.22	0.7	0.28	0.18	43 ± 16/161 ± 8

Table SI2. SAED patterns analysis of the sample mechanochemical treated by ball milling for 6 hours. The measured d-spacings are reported for the bCC powders and the geogenic one. The d-spacing of aragonite and calcite are reported for comparison.

Geo-$CaCO_3$ (Å)	Oyster (Å)	Scallop (Å)	Clam (Å)	aragonite (Å)	calcite (Å)
3.84	3.80	3.82	3.37	3.39	3.86
3.00	3.01	3.36	2.70	2.70	3.03
2.47	2.75	2.99	2.37	2.37	2.49
2.32	2.40	2.46	1.97	1.98	2.28

Table SI3. Percentage of $CaCO_3$ polymorphs and crystallite size of geogenic calcium carbonate, oyster shell, scallop shell and clam shell powder after different aging times in diverse solvents. The instrumental error is reported.

**environment**	**Aging time**	**Calcite** (wt. %)	**Aragonite** (wt. %)$^{\$}$	**ACC** (wt. %)$^{\#}$	$\mathbf{d}_{(104)}/\mathbf{d}_{(111)}$ (nm)
**geo $CaCO_3$**					
N_2	10 days	74 ± 2	/	26 ± 2	12.18 ± 0.86
	20 days	84 ± 2	/	16 ± 2	13.61 ± 0.56
	30 days	85 ± 2	/	15 ± 2	15.44 ± 0.43
EtOH	15 min	90 ± 2	/	10 ± 2	12.14 ± 0.38
	30 min	90 ± 2	/	10 ± 2	12.56 ± 0.49
	120 min	97 ± 2	/	3 ± 2	16.00 ± 3.10
H_2O	5 min	100 ± 2	/	0 ± 2	21.60 ± 0.42
	30 min	100 ± 2	/	0 ± 2	23.43 ± 0.65
	120 min	100 ± 2	/	0 ± 2	25.38 ± 0.74
**Oyster shell**					
N_2	10 days	43 ± 2	18 ± 2	39 ± 2	8.41 ± 0.49/14.20 ± 1.20
	20 days	53 ± 2	27 ± 2	20 ± 2	13.59 ± 0.46/20.17 ± 0.91
	30 days	68 ± 2	27 ± 2	5 ± 2	17.55 ± 0.46/19.82 ± 0.87
EtOH	15 min	51 ± 2	22 ± 2	27 ± 2	7.99 ± 0.17/14.00 ± 1.20
	30 min	47 ± 2	26 ± 2	27 ± 2	9.69 ± 0.18/15.43 ± 0.93
	120 min	51 ± 2	26 ± 2	23 ± 2	12.87 ± 0.21/18.56 ± 0.84
H_2O	5 min	74 ± 2	23 ± 2	4 ± 2	29.38 ± 0.73/19.02 ± 0.60
	30 min	84 ± 2	13 ± 2	3 ± 2	30.05 ± 0.39/19.80 ± 1.20
	120 min	89 ± 2	11 ± 2	0 ± 2	38.24 ± 0.91/23.70 ± 1.40
**Scallop shell**					
N_2	10 days	48 ± 2	8 ± 2	44 ± 2	7.79 ± 0.17/14.80 ± 1.70
	20 days	46 ± 2	19 ± 2	35 ± 2	8.89 ± 0.19/13.40 ± 1.10
	30 days	49 ± 2	23 ± 2	28 ± 2	12.36 ± 0.46/20.20 ± 1.00
EtOH	15 min	42 ± 2	14 ± 2	44 ± 2	8.68 ± 0.69/13.10 ± 1.50
	30 min	48 ± 2	19 ± 2	33 ± 2	10.17 ± 0.51/17.30 ± 1.30
	120 min	57 ± 2	23 ± 2	20 ± 2	13.58 ± 0.41/18.80 ± 1.00
H_2O	5 min	82 ± 2	14 ± 2	3 ± 2	27.83 ± 0.61/17.84 ± 0.88
	30 min	89 ± 2	9 ± 2	2 ± 2	37.30 ± 1.00/18.70 ± 1.50
	120 min	91 ± 2	8 ± 2	1 ± 2	35.05 ± 0.84/21.30 ± 1.60
**Clam shell**					
N_2	10 days	36 ± 2	26 ± 2	38 ± 2	6.73 ± 0.38/12.08 ± 0.59
	20 days	38 ± 2	42 ± 2	20 ± 2	12.39 ± 0.81/16.53 ± 0.55
	30 days	43 ± 2	46 ± 2	11 ± 2	16.06 ± 0.64/16.76 ± 0.42
EtOH	15 min	35 ± 2	29 ± 2	36 ± 2	6.65 ± 0.18/11.33 ± 0.54
	30 min	35 ± 2	36 ± 2	29 ± 2	8.81 ± 0.44/14.31 ± 0.54
	120 min	47 ± 2	50 ± 2	3 ± 2	14.93 ± 0.72/16.08 ± 0.47
H_2O	5 min	55 ± 2	43 ± 2	2 ± 2	25.56 ± 0.75/25.90± 1.70
	30 min	65 ± 2	33 ± 2	2 ± 2	32.85 ± 1.00/26.90 ± 1.80
	120 min	66 ± 2	33 ± 2	1 ± 2	32.08 ± 0.87/27.40 ± 1.70

$^{\$}$ Percentage of crystalline phases. $^{\#}$ Percentage of ACC in the particles.
